# Leveraging expression quantitative trait loci information in single-cell resolution to identify cell-specific genes for Basal cell carcinoma

**DOI:** 10.1371/journal.pone.0354887

**Published:** 2026-08-04

**Authors:** Min Du, Wenyan Zhu, Shiqi Wang, Rong Wang, Xiuqi Li

**Affiliations:** 1 Department of Plastic and Cosmetic surgery and Burns, Qilu Hospital of Shandong University, Jinan, China; 2 Department of Epidemiology, Center for Global Health, School of Public Health, Nanjing Medical University, Nanjing, China; West Bengal University of Animal and Fishery Sciences, INDIA

## Abstract

**Background:**

Basal cell carcinoma (BCC), the most common skin cancer, is driven by UV-induced DNA damage and shaped by immune surveillance. Although GWAS has identified over 140 risk loci, their cell-type-specific effects remain obscured by tissue-level averaging.

**Methods:**

We integrated BCC GWAS summary statistics from a UK-based cohort (17,416 cases, 375,455 controls) with single-cell expression quantitative trait locus (sc-eQTL) data from 12 immune cell types in the OneK1K resource. Using the OTTERS framework combined with ACAT-O, we performed single-cell transcriptome-wide association analysis (scTWAS); bulk TWAS using GTEx whole blood served as a conventional tissue-averaged benchmark for comparison, rather than a definitive gold standard. Functional enrichment was conducted via Gene Ontology (GO).

**Results:**

Bulk TWAS using GTEx whole blood identified 35 BCC-associated genes (FDR < 0.05), including *MC1R* and *CASP8*. In contrast, single-cell transcriptome-wide association study (scTWAS) across 12 OneK1K cell types revealed 207 non-redundant susceptibility genes, predominantly in CD4_ET_, MONO_C_, and B_IN_. Functional enrichment uncovered cell-type-specific programs: MHC class II antigen presentation (CD4_ET_), PRR-mediated innate immunity (MONO_C_), and pro-inflammatory secretion (B_IN_)—all absent in bulk results.

**Conclusion:**

Our exploratory scTWAS using healthy donor PBMC-derived eQTLs uncovered cell-type-specific BCC associations missed by bulk analyses, providing hypothesis-generating insights into immune-related genetic effects in BCC. This underscores how single-cell resolution can overcome signal dilution from cellular heterogeneity, though validation in tumor-derived immune populations is warranted.

## 1. Introduction

Basal cell carcinoma (BCC) is the most common form of skin cancer in the United Kingdom and among the most prevalent human malignancies worldwide [1]. It accounts for approximately 10–15% of all carcinomas in women and up to 20% in men, with 80–85% of lesions arising in the head and neck region [2]. The current pathogenic model posits that cumulative ultraviolet (UV) radiation exposure induces characteristic UV-signature mutations, leading to DNA damage in epidermal basal cells [3]. Individuals with fair skin, light or red hair, and pale eye color are at substantially elevated risk, largely due to reduced melanin-mediated photoprotection against UV photons and reactive oxygen species [4,5].

Genome-wide association studies (GWAS), transcriptome-wide association studies (TWAS), plasma proteomics have significantly improved our knowledge regarding the genetic, genomic and proteomic architecture of BCC [6–10]. Adolphe *et al.* and Seviiri *et al.* identified risk polymorphisms at key susceptibility loci—including *MC1R* and *IRF4* — with subsequent large-scale meta-analyses confirming over 140 expression and methylation quantitative trait loci (eQTLs/mQTLs) associated with BCC risk, highlighting roles for pigmentation, UV response, and immune regulation in BCC pathogenesis [9]. Single-cell RNA sequencing (scRNA-seq) has revealed profound intratumoral heterogeneity and distinct transcriptional cell states in infiltrative BCCs, including tumor-stroma interface subpopulations marked by dysregulation of Hedgehog signaling (e.g., *PTCH1* loss) and enriched for invasive signatures [11]. However, these data alone cannot link germline genetic variation to context-specific gene regulation. Recent advances in single-cell expression quantitative trait locus (sc-eQTL) mapping now enable direct association of genotypes with gene expression at single-cell resolution, as demonstrated in large-scale immune and cancer atlases including OneK1K [12] and TenK10K [13]. Although neither atlas was derived from BCC tissues, both profile immune cell populations—such as T cells, NK cells, and monocytes—that are known to infiltrate the BCC tumor microenvironment and contribute to its immunological landscape [14]. Thus, these resources provide a relevant proxy for interrogating immune-mediated genetic effects in BCC. Powell *et al.* analyzed over one million immune cells from 1,000 individuals and demonstrated that the majority of eQTLs are active only in specific cell states or subtypes—a finding masked in bulk tissue analyses—thereby establishing a foundational framework for interpreting non-coding disease variants [12]. Importantly, sc-eQTL studies have shown that disease-associated SNPs often regulate gene expression exclusively in rare or context-dependent cell populations [15,16]. For instance, Chen *et al.* identified thousands of cell-type-restricted sc-eQTLs in human tumors, with strong enrichment near transcription start sites and dramatic variability in detection across lineages [15]. Recently, the single-cell transcriptome-wide association study (scTWAS) Atlas was launched as a comprehensive knowledge base for single-cell transcriptome-wide association studies, integrating precomputed scTWAS results across multiple cell types and complex traits [17]. While this resource provides valuable insights into cell-type-specific genetic architecture, it currently lacks coverage of skin malignancies such as BCC and does not include specialized tumor-infiltrating immune or malignant cell states relevant to BCC biology. Moreover, as a static repository built upon existing sc-eQTL references, it cannot accommodate newly released GWAS summary statistics. Consequently, resources like the latest BCC meta-analytic GWAS data [10] cannot be used for customized, up-to-date scTWAS interrogation. Therefore, a dedicated analysis leveraging state-of-the-art frameworks like OTTERS remains essential to uncover BCC-associated genes at cellular resolution.

**Fig 1:**
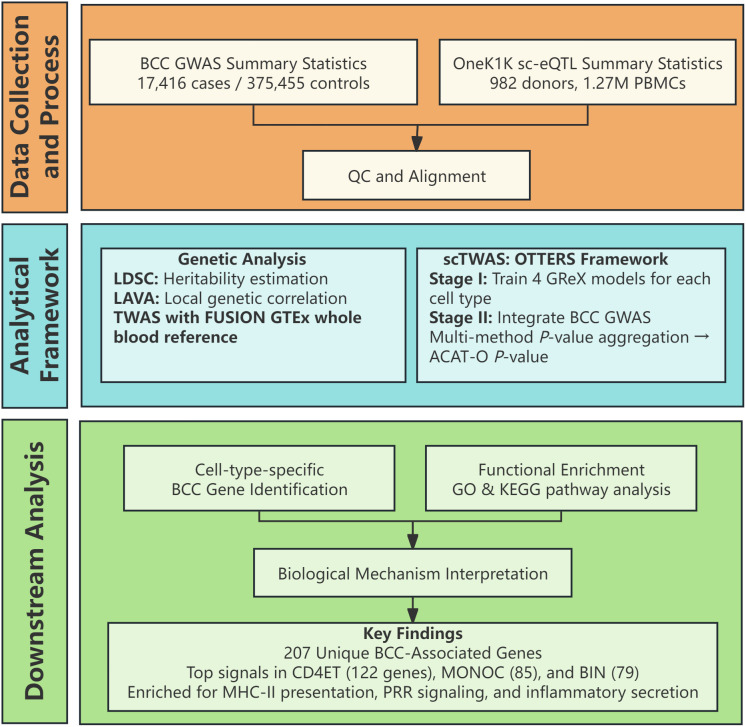
Single-cell resolution eQTL-based framework for identifying cell-type-specific risk genes in BCC compared with traditional bulk tissue TWAS approach.

Overall, existing studies have defined significant loci and genes through in- and cross-ancestry GWAS and post-GWAS analyses [18], as well as cell-type-specific transcriptional programs by scRNA-seq in BCC [15,19]. Current population-level genetic studies of BCC primarily rely on bulk tissue or germline summary statistics, which overlook the profound cellular heterogeneity of the tumor microenvironment and may obscure cell-type-specific genetic effects. Single-cell transcriptomic studies in BCC have revealed distinct malignant and immune subpopulations with strong biological signals, but are often limited by small sample sizes [17]. These drawbacks make findings at the cellular mechanism level difficult to generalize or integrate into population-scale genetic inference. To address this issue, leveraging the summary statistics of sc-eQTL reference panels, we applied a single-cell-resolution TWAS framework to prioritize BCC-associated genes [20]. Enrichment analysis across different cell types implicated these genes in distinct biological pathways (e.g., Hedgehog signaling dysregulation, UV-induced DNA damage response, and immune modulation), providing deeper pathophysiological insights into BCC, which was complemented by a comparative analysis using traditional TWAS based on whole-blood eQTLs from GTEx. (Fig 1).

We acknowledge that the OneK1K sc-eQTL reference is derived from healthy donor peripheral blood rather than BCC tumor tissue. Therefore, our analyses represent an in silico prediction of cell-type-specific genetic effects in circulating immune cells that may be relevant to BCC, rather than a direct measurement of the tumor microenvironment.

## 2. Materials and Methods

### 2.1. Data resource

We received summary statistics for BCC from a UK-based genome-wide association study, which included 17,416 cases and 375,455 controls of Western European ancestry [21]. Following established protocols [22–24], we implemented a stringent SNP quality control (QC) pipeline. Specifically, we removed variants with missing effect alleles or standard errors and retained only those present in the HapMap3 (hm3) reference panel (https://www.nature.com/articles/nature09298). We maintained 1,229,997 high-quality SNPs. This improved dataset was then utilized to conduct scTWAS analysis to detect cell-type-specific genetic correlations with BCC. Ambiguous palindromic SNPs (A/T and C/G SNPs) were removed prior to allele harmonization, following standard practice in TWAS and GWAS harmonization pipelines.

Based on the OneK1K cohort, we used scRNA-seq data from about 1.27 million peripheral blood mononuclear cells (PBMCs) collected from 982 healthy donors [12]. The original dataset underwent rigorous quality control: SNPs with call rate < 95%, minor allele frequency (MAF) < 1%, or Hardy-Weinberg equilibrium *P* < 1 × 10^−6^ were excluded prior to imputation [12]. To ensure compatibility with the BCC GWAS summary statistics and enable accurate functional interpretation in the current genomic reference, we converted the OneK1K cis-eQTL mappings from GRCh37 to GRCh38 using liftOver (https://genome.ucsc.edu/cgi-bin/hgLiftOver). Following coordinate conversion, we harmonized alleles and effect directions between the eQTL and GWAS datasets. This alignment yielded 1,026,359 shared SNPs, which served as the foundation for genetically regulated expression (GReX) modeling in the OTTERS framework. We analyzed eQTL data from 12 immune cell subtypes: CD4^+^ effector memory T cells (CD4_ET_), CD4^+^ naive and central memory T cells (CD4_NC_), CD4^+^ T cells expressing SOX4 (CD4_SOX4_), CD8^+^ naive and central memory T cells (CD8_NC_), CD8^+^ T cells with expression of S100B (CD8_S100B_), CD8^+^ effector memory T cells (CD8_ET_), Memory B cells (B_Mem_), Immature and naive B cells (B_IN_), Plasma cells (Plasma), Classical monocytes (Mono_C_), Nonclassical monocytes (Mono_NC_), and Dendritic cells (DC).

### 2.2. Genetic Analysis for BCC

We performed the post-GWAS analysis of BCC in two levels. At the trait level, we first applied LD Score Regression (LDSC; v1.0.1) [25] to the BCC GWAS summary statistics to estimate SNP-based heritability and assess potential confounding factors such as population stratification and cryptic relatedness. Next, we performed local heritability partitioning using Local Analysis of [co]Variant Association (LAVA; v0.1.0) [26]. LAVA jointly observed and latent genetic effects while accounting for LD and polygenicity across the genome. The analysis partitioned the genome into non-overlapping LD blocks based on the 1000 Genomes Project EUR reference panel, and each locus was tested for significant enrichment of BCC heritability beyond the null expectation. Empirical *P*-values were derived via permutation, and significance was defined after Bonferroni correction (*P* = 0.05/ number of loci). At the molecular level, we conducted a TWAS using the FUSION framework [27]. FUSION integrates GWAS summary statistics with precomputed expression prediction models derived from GTEx whole blood to test for associations between genetically regulated gene expression and BCC risk. The analysis automatically restricts SNPs present in both the GWAS input and the expression weights, accounting for local linkage disequilibrium using a 1000 Genomes Project European reference panel. Gene-level *P* values were adjusted for multiple testing via the Benjamini–Hochberg procedure, and results with false discovery rate (FDR) < 0.05 were considered significant.

### 2.3. scTWAS Analysis

We performed scTWAS using the OTTERS framework [20], which integrates multiple expression imputation models to enhance robustness in gene–trait association testing. Critically, we independently implemented Stage I of OTTERS—the computationally intensive step of training gene-level GReX imputation models across all autosomal genes. Specifically, for each of the 12 immune cell types profiled in the OneK1K sc-eQTL resource, we trained four distinct GReX models per gene using lassosum, SDPR, PRS-CS, and a frequentist P + T baseline, as implemented in OTTERS. These models were derived from summary-level cis-eQTL statistics provided by the OneK1K consortium, combined with an ancestry-matched LD reference panel constructed from 503 European-ancestry individuals in the 1000 Genomes Project.

To ensure biological relevance and computational consistency, gene annotations were obtained from GENCODE v41 (https://www.gencodegenes.org/), and cis-regions were defined as ±1 Mb around each gene’s transcription start (GeneStart) and end (GeneEnd) sites. All model training was performed on high-performance computing clusters, requiring substantial computational resources due to the scale of genome-wide modeling across multiple cell types and methods. The resulting eQTL weight files, which comprise over 200,000 gene-cell-method combinations, have been made publicly available via Figshare (DOI: 10.6084/m9.figshare.30632813) to support future scTWAS applications in pregnancy-related or immune-mediated traits.

In Stage II, we applied these pre-trained models to the BCC GWAS summary statistics to impute GReX and compute gene-level association Z-scores for each method and cell type. Finally, we aggregated the four method-specific TWAS *P* values per gene using the ACAT-O omnibus test to produce a single [20], robust association statistic that leverages complementary strengths of diverse imputation strategies. Following the OTTERS framework, we applied the default quality control thresholds, retaining models with cross-validation *R*^*2*^ > 0.01 and cross-validation correlation *P* < 0.05. The number of genes retained for Stage II analysis in each cell type is reported in Supplementary S3 Table. For multiple testing correction, we applied the Benjamini-Hochberg FDR procedure across all gene-cell type tests combined (i.e., jointly across all cell types), and associations with FDR < 0.05 were considered significant.

### 2.4. Enrichment Analysis

We used *clusterProfiler* package (v4.10.1) to conduct functional enrichment analysis on gene sets resulting from ACAT results [28–30]. This program allows for over-representation analysis (ORA) of GO and KEGG pathways, as well as comparative display of enriched biological topics across gene clusters. We utilized clusterProfiler’s enrichGO and enrichKEGG algorithms with default parameters to discover significantly enriched phrases, which were then visualized using the built-in dot and bubble plots. Gene annotations were based on human genome databases, which aligned with the package’s recommended process for human data.

### 2.5. Ethics Statement

This study is a secondary analysis of publicly available or collaboratively shared, de-identified summary-level data. The skin cancer GWAS data were derived from cohorts approved by the University of Queensland Human Research Ethics Committee (2011001173) and the UK Biobank (North West MREC 11/NW/0382) [21]. The OneK1K sc-eQTL data were collected with ethics approval from the Tasmania Health and Medical Human Research Ethics Committee (H0012902) and participant written informed consent. Given that this work involved no interaction with human subjects and only used anonymized data, it was exempt from further ethical review by the Ethics Committee of Nanjing Medical University.

### 2.6. Software

All statistical analyses and visualizations were performed in R (version 4.3.2; https://www.R-project.org/).

## 3. Results

### 3.1. Post-GWAS analysis for BCC

**Fig 2:**
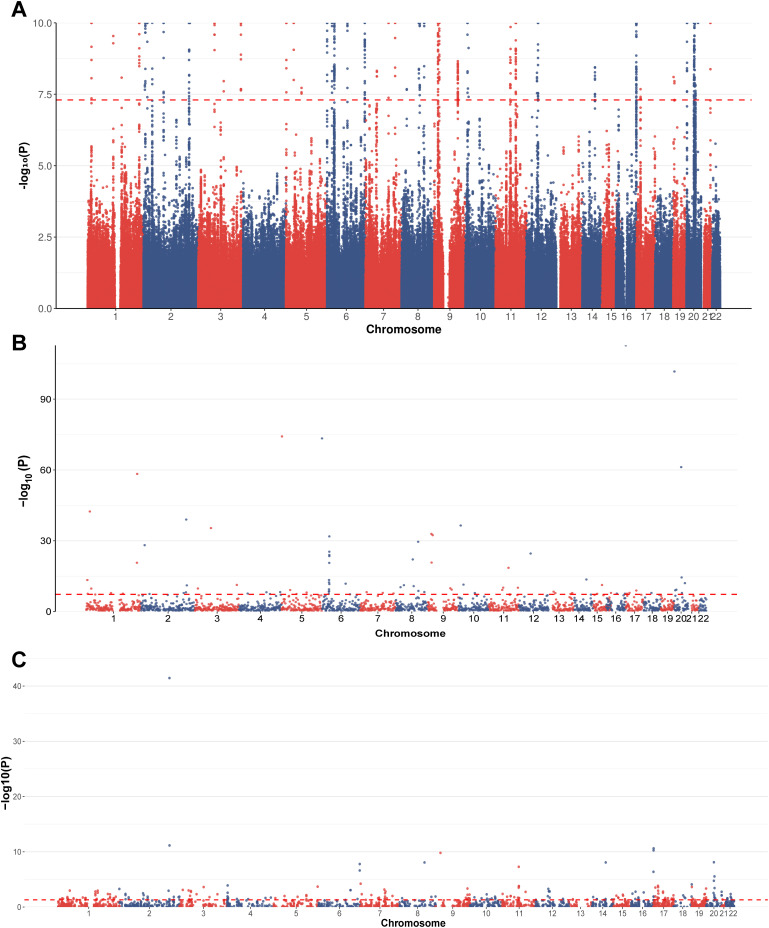
Summary for the post-GWAS analysis of BCC. (A) Manhattan plot for summary statistics; (B) Manhattan plot for local heritability; (C) Manhattan plot for FUSION.

Based on LDSC, the observed SNP-based heritability of BCC was 0.0360 (standard error [SE] = 0.0036, *P* < 0.001; Fig 2A). To further dissect the genomic architecture, LAVA identified 223 genomic regions significantly enriched for BCC heritability after Bonferroni correction (α = 0.05/ number of loci), with the strongest signals on chromosomes 16 (e.g., chr16: 89.0–90.2 Mb, local h^2^ = 0.0020, *P* = 1.59 × 10⁻^113^) and chromosome 20 (e.g., chr20: 1.8–2.7 Mb, local h^2^ = 0.0019, *P* = 1.87 × 10^-102^). (Fig 2B, S1 Table). Using TWAS with GTEx whole blood gene expression models, we identified 35 genes significantly associated with BCC. After false discovery rate (FDR) correction (q < 0.05), the top five most significant associations were: *CASP8* (TWAS.Z = -13.61, TWAS.*P* = 3.50 × 10^-42^), *STRADB* (TWAS.Z = -6.86, TWAS.*P* = 7.07 × 10^-12^), *MC1R* (TWAS.Z = -6.68, TWAS.*P* = 2.41 × 10^-11^), *SPATA33* (TWAS.Z = -6.56, TWAS.*P* = 5.48 × 10^-11^), and *CDKN2B* (TWAS.Z = -6.40, TWAS.*P* = 1.56 × 10^-10^) (Fig 2C, S2 Table).

### 3.2. sc-eQTL

**Fig 3:**
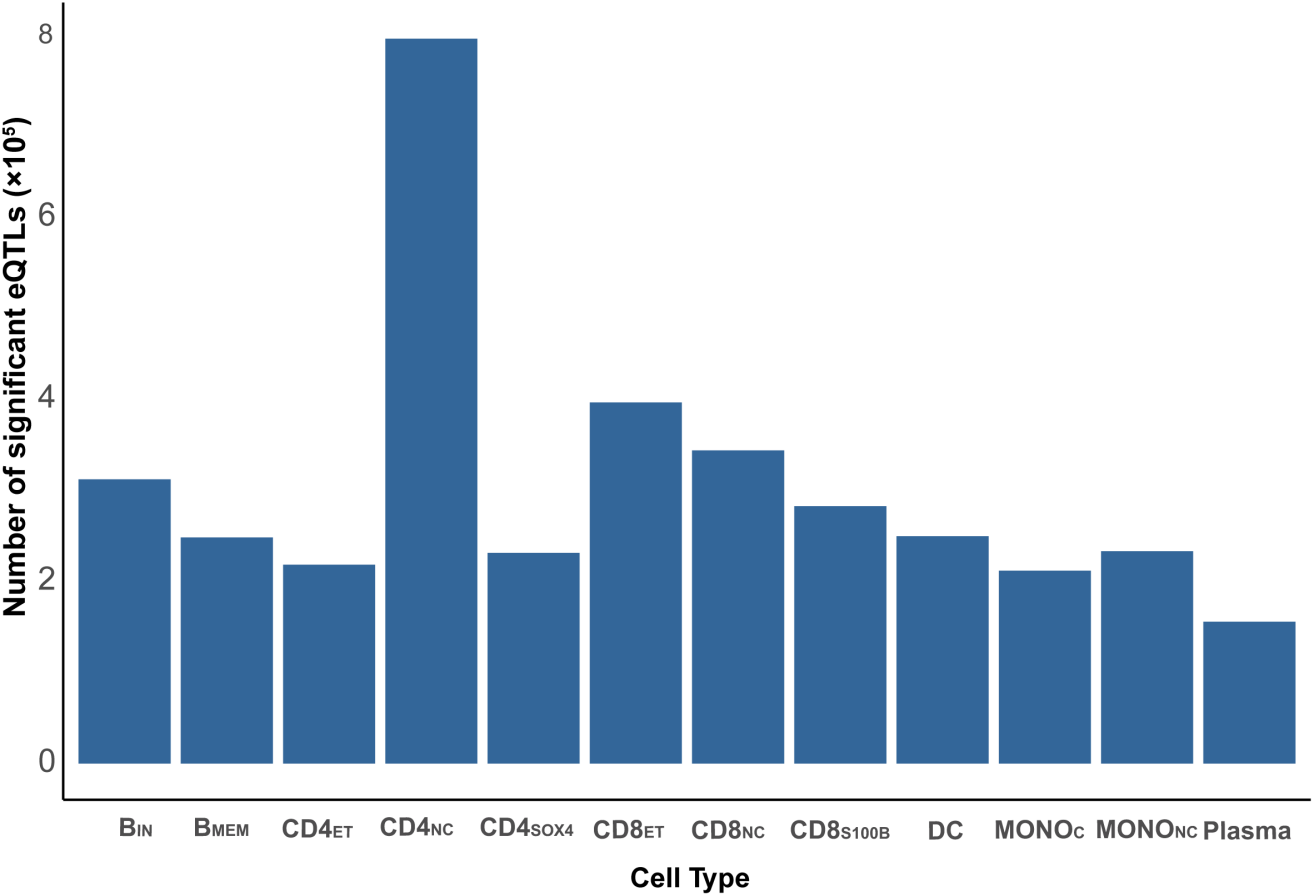
Bar plot indicates the significant eQTL number across each cell type.

We quantified the number of significant cis-sc-eQTLs (FDR < 0.05) across 12 immune cell types in the OneK1K cohort. CD4_NC_ exhibited the highest regulatory complexity, with 795,929 significant eQTLs—more than twice that of other major lineages. CD8_ET_ and CD8_NC_ followed, with 396,523 and 343,885 eQTLs, respectively. In contrast, plasma cells showed the lowest number (155,713), while B cells, dendritic cells, and monocyte subsets displayed intermediate levels (Fig 3). This pronounced heterogeneity underscores the cell-type-specific nature of genetic regulation and positions CD4^+^ T cells as a key cellular context for downstream BCC association analysis.

**Fig 4:**
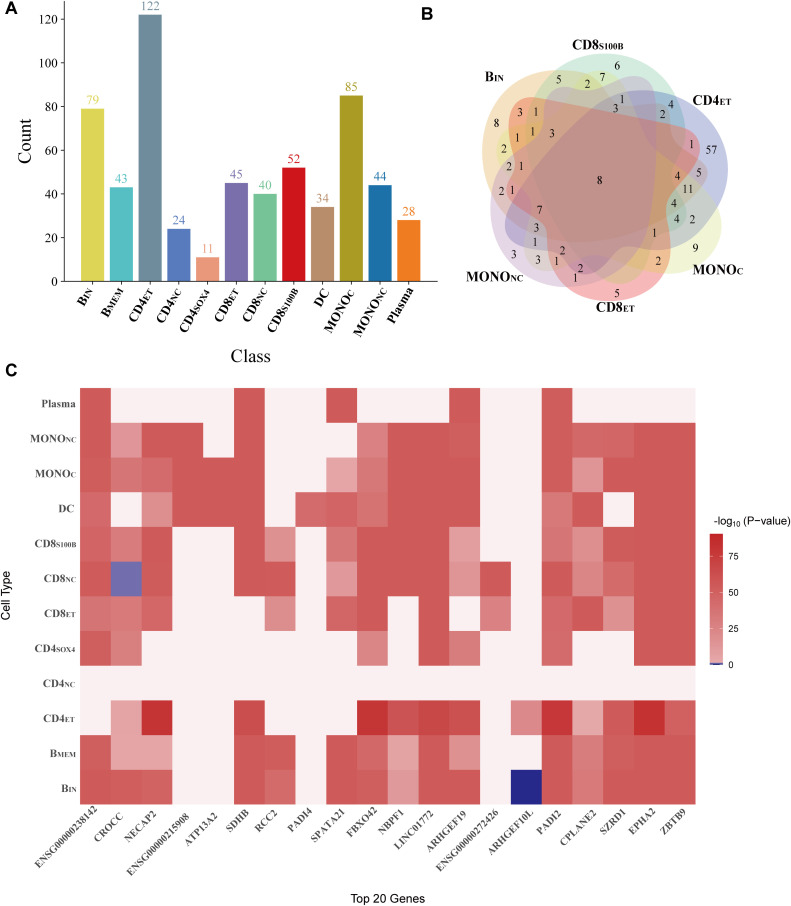
Summary for the sc-QTL analysis. (A) Bar plot indicates the number of significant genes across each cell type. (B) Heatmaps show the P value for the top 20 genes. (C) Venn diagram representing six cell populations.

We performed single-cell transcriptome-wide association analysis (sc-TWAS) by integrating OTTERS with ACAT across 12 immune cell types from the OneK1K cohort. After FDR correction (q < 0.05), a total of 607 significant gene–cell type (S3 Table) associations were detected; however, these corresponded to 207 unique genes after removing duplicates. The number of significant genes differed markedly across cell types: CD4ET exhibited the highest burden (122 genes), followed by MONO_C_ (85), B_IN_ (79), and CD8_S100B_ (52), while other subsets such as CD4_SOX4_ (11) and Plasma (28) showed more limited signals (Fig 4A). Notably, a small subset of genes (e.g., LOC105376805, ENSG00000215908) showed near-constant P-values across all cell types (varying by < 1 × 10^−5^), suggesting these signals may reflect algorithmic artifacts or LD hitchhiking rather than genuine cell-type-specific biology. These loci were excluded from downstream functional enrichment analyses. Importantly, the number of significant genes per cell type and the top enriched GO terms remained essentially unchanged after their exclusion (data not shown), confirming that our main biological conclusions are not driven by these suspected artifacts. Therefore, we focused subsequent interpretation on genes with variable, cell-type-restricted signals. Among the top 20 genes ranked by ACAT *p*-value across all cell types, many displayed restricted or enriched associations—such as strong signals in myeloid lineages (MONO_C_, DC) or adaptive immune compartments (B_MEM_, CD4_ET_)—as visualized in the heatmap (Fig 4B). Focusing on the six cell types with the most significant BCC-associated genes—CD4_ET_, MONO_C_, B_IN_, CD8_S100B_, CD8_ET_, and MONO_NC_—Venn diagram revealed both shared and cell-type-specific signals. Notably, genes such as *ARHGAP18*, *CNOT7*, and *L3MBTL3* were co-significant in MONO_C_, CD4_ET_, CD8_ET_, and B_IN_, suggesting core roles in antigen presentation or interferon response (Fig 4C). Together, these results highlight distinct genetic regulatory programs across innate and adaptive immune subsets in the context of BCC.

### 3.3. Enrichment analysis

**Fig 5:**
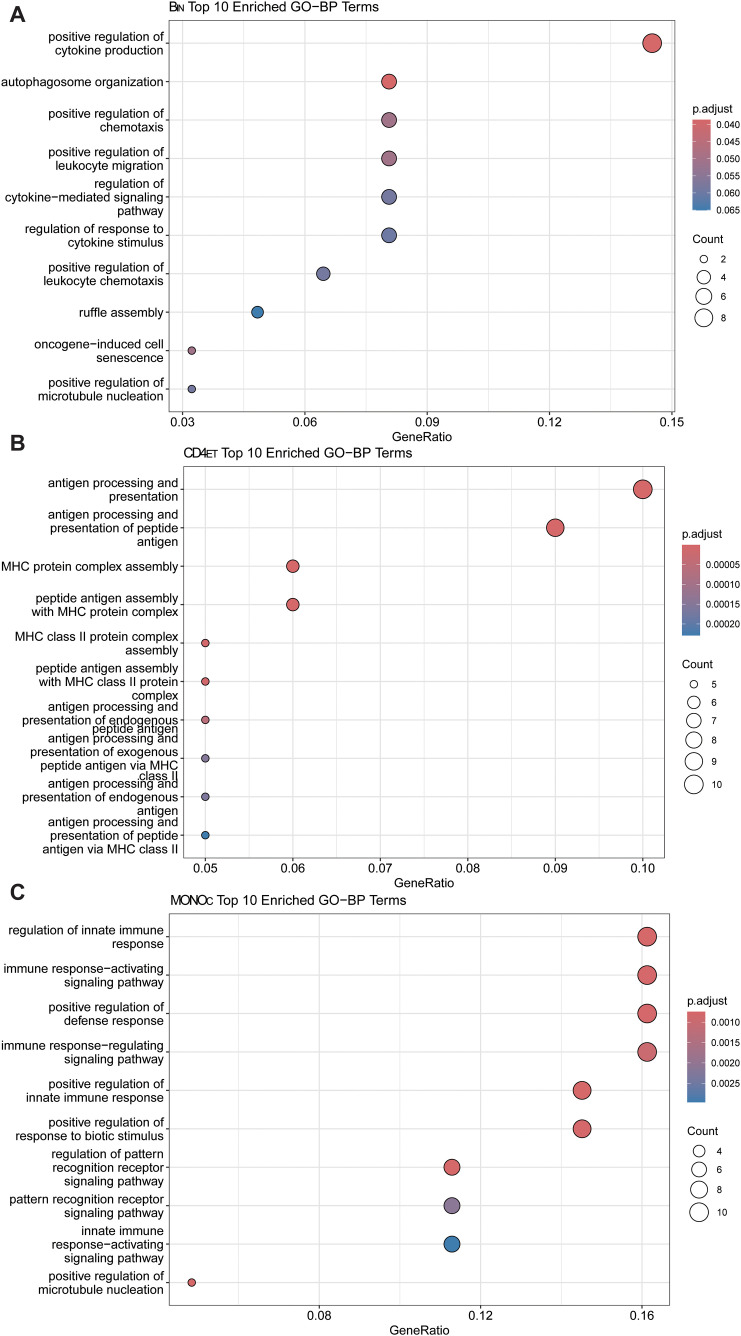
Gene Ontology enrichment of BCC-associated genes in B_IN_, CD4_ET_ and MONO_C_ (FDR < 0.05).

We performed functional enrichment analysis on the significant BCC-associated genes identified by sc-TWAS in the three immune cell types showing the strongest genetic signals: B_IN_, CD4_ET_, and MONO_C_ (Fig 5). In CD4_ET_, the top enriched Gene Ontology terms were overwhelmingly centered on antigen processing and presentation, including “antigen processing and presentation of peptide antigen via MHC class II” (FDR = 5.0 × 10^−5^), “MHC class II protein complex assembly” (FDR = 1.0 × 10^−4^), and related terms involving peptide loading and MHC complex formation—highlighting a critical role for CD4^+^ effector T cells in coordinating adaptive immune recognition in BCC. In contrast, MONO_C_ displayed a robust signature of innate immune activation, with significant enrichment for “pattern recognition receptor signaling pathway” (FDR = 0.0021), “regulation of innate immune response” (FDR = 7.4 × 10^−4^), “positive regulation of defense response” (FDR = 7.7 × 10^−4^), and “innate immune responseactivating signaling pathway” (FDR = 0.0030), consistent with a myeloid-driven inflammatory response. Notably, B_IN_ (immature and naive B cells) showed enrichment for processes involved in immune modulation and cellular stress, such as ‘positive regulation of cytokine production’ (FDR = 0.0386), ‘autophagosome organization’ (FDR = 0.0386), and ‘positive regulation of leukocyte chemotaxis’ (FDR = 0.0506), and “oncogene-induced cell senescence” (FDR = 0.065). Together, these distinct functional profiles highlight cell-type-specific genetic programs in circulating immune cells that may be relevant to BCC pathogenesis. However, we emphasize that these findings are derived from healthy donor PBMCs and require validation in tumor-associated immune populations. Additional Gene Ontology enrichment results for the remaining immune cell types are presented in S1-S3 Figs.

These three cell types were selected for visualization as they harbored the highest number of significant TWAS-discovered genes.

We observed a limited overlap between bulk TWAS and scTWAS: bulk TWAS identified 35 genes (including well-established BCC loci such as *MC1R* and *CASP8*), while scTWAS identified 207 non-redundant genes, with only two genes (SH3YL1 and *L3MBTL3*) significant in both analyses. However, this limited concordance should be interpreted cautiously, as differences between the two approaches—including distinct eQTL reference panels (GTEx whole blood vs. OneK1K PBMC subtypes), methodological differences in imputation models, and statistical power—may contribute to the observed discordance, rather than solely reflecting cell-type resolution. Nevertheless, the broader set of genes identified by scTWAS and their cell-type-restricted patterns suggest that single-cell resolution can complement bulk analyses by revealing context-specific genetic effects that are averaged out in tissue-level data. Specifically, we note that *L3MBTL3*, a gene identified in both bulk and scTWAS, belongs to the Polycomb family and is implicated in chromatin remodeling and cell cycle regulation [31]. Furthermore, *SH3YL1*, another consensus gene, plays a critical role in regulating the actin cytoskeleton and endocytosis, which are fundamental processes for immune cell function [32]. These biological functions provide a plausible mechanistic link to BCC pathogenesis, supporting the validity of our findings despite the lack of external datasets.

## 4. Discussion

In this study, we applied sc-TWAS to dissect the cell-type-specific genetic architecture of BCC. While conventional bulk TWAS using GTEx whole blood identified 35 significant susceptibility genes—including well-established pigmentation (*MC1R*) and apoptosis (*CASP8*) loci—our integrative sc-TWAS approach, combining OTTERS with ACAT across 12 immune cell types from the OneK1K cohort, revealed a substantially more refined landscape. We detected 607 significant gene-cell type associations at FDR < 0.05, corresponding to 207 non-redundant BCC-associated genes. Notably, these signals were highly unevenly distributed across cell types, with the three lineages harboring the greatest number of significant associations—CD4_ET_, MONO_C_, and B_IN_—accounting for the majority of discoveries. This highlights how single-cell resolution exposes biologically coherent regulatory programs that are obscured by cellular averaging in bulk tissue analyses [17,33].

The most striking enrichment was observed in CD4ET cells, where BCC-associated genes were overwhelmingly involved in MHC class II-mediated antigen processing and presentation. Top terms included “peptide antigen assembly with MHC class II protein complex” and “MHC class II protein complex assembly”, implicating genes such as HLA-DRA, CD74, and HLA-DM family members. This suggests that circulating CD4^+^ effector T cells from healthy donors exhibit genetic programs related to MHC class II antigen presentation, which may be relevant to immune surveillance in BCC [34]. However, we caution that these findings are derived from peripheral blood and may not fully capture the functional state of tumor-infiltrating CD4^+^ T cells in BCC lesions [35]. Furthermore, we acknowledge that this enrichment may be influenced by the complex linkage disequilibrium structure of the HLA region, as well as potential technical artifacts such as residual doublet contamination or ambient RNA in the single-cell data, which could disproportionately affect CD4^+^ T cell annotations. Thus, while the MHC-II enrichment is biologically plausible, it should be interpreted as hypothesis-generating and warrants validation in BCC-derived tumor-infiltrating immune populations.

Concurrently, classical monocytes (MONO_C_) exhibited a robust signature of innate immune activation, with significant enrichment for “pattern recognition receptor signaling”, “positive regulation of innate immune response”, and “defense response”. These pathways are hallmarks of myeloid cell sensing of damage-associated molecular patterns (DAMPs), which may be relevant to the inflammatory response triggered by UV-damaged keratinocytes in BCC [36]. While our findings in circulating classical monocytes align with histopathological observations of monocytic infiltrates in aggressive BCC subtypes, we note that the regulatory state of circulating monocytes may differ from that of tumor-infiltrating monocytes. Therefore, these results should be interpreted as hypothesis-generating and require validation in BCC-derived myeloid populations [37].

In B_IN_ cells, we observed enrichment for genes involved in cytokine production and leukocyte chemotaxis. While these findings are derived from circulating B cells rather than tumor-infiltrating cells, they raise the hypothesis that B cell-mediated signaling might contribute to immune recruitment in BCC. However, we caution that the OneK1K dataset originates from healthy peripheral blood; therefore, these results reflect genetic regulatory programs in circulating immune cells and should not be directly extrapolated to the BCC tumor microenvironment without further validation.

Our sc-TWAS and functional enrichment results highlight cell-type-specific genetic associations in immune cells: CD4_ET_ cells show enrichment for MHC class II-mediated antigen presentation, MONO_C_ exhibits a pattern recognition receptor-dependent innate inflammatory signature, and B_IN_ (circulating immature B cells) displays enrichment for cytokine production and chemotaxis-related pathways. While these findings provide insights into immune-related genetic mechanisms potentially relevant to BCC, they are derived from healthy donor PBMCs and require validation in tumor-associated immune populations.

We also note that direct comparison between scTWAS and bulk TWAS is complicated by differences in eQTL reference construction; thus, the additional scTWAS signals should be interpreted as hypothesis-generating rather than definitive.

Together, these cell-type-specific associations provide a hypotheses-generating framework for understanding how BCC-associated GWAS variants may influence immune-related pathways. However, given that our analyses are based on healthy donor PBMCs, future studies using BCC-derived single-cell eQTL data will be necessary to confirm whether these programs operate within the tumor microenvironment.

We also note that our scTWAS analysis restricted cis-eQTL modeling to HapMap3 variants, following standard TWAS practice as implemented in FUSION and common PrediXcan pipelines, where the LD reference panel is similarly constrained to ensure reliable imputation and consistent allele harmonization. However, we acknowledge as a limitation that our framework does not incorporate annotation-aware methods with expanded variant sets, such as SBayesRC, which leverage functional genomic annotations to improve statistical power and may recover additional susceptibility loci not detectable under our current approach. Despite these insights, several limitations exist. First, the OneK1K sc-eQTL reference was derived from peripheral blood of healthy donors, which may not reflect the transcriptional states of tumor-infiltrating immune cells in sun-exposed skin or BCC lesions. Second, our functional interpretation of B_IN_ cells—which are circulating immature B cells—should not be conflated with tumor or stromal biology; these results reflect genetic programs in healthy peripheral blood immune cells and serve as a baseline for future studies of tumor-associated B cells. Third, while our BCC GWAS is one of the largest to date, power remains limited for rare variants or cell-type-specific effects. Fourth, sc-TWAS infers gene-trait associations but cannot establish causality. Future work should integrate CRISPR screens in relevant cell types to validate top candidates like HLA-DRA or IRF family members, ideally using BCC-derived single-cell eQTL data when available. Fifth, several top-ranked associations in our scTWAS (e.g., ENSG00000238142) exhibited near-identical P-values across all cell types, suggesting potential algorithmic artifacts or LD-driven inflation rather than genuine cell-type-specific regulation. Future studies should validate these loci experimentally before biological interpretation. Sixth, although the OneK1K dataset underwent rigorous doublet removal, we cannot completely exclude residual doublet contamination in CD4_ET_ cells; future validation using flow-sorted pure populations is warranted. Seventh, due to the lack of BCC-derived sc-eQTL references and publicly available BCC single-cell datasets with genotype information, external validation of the identified gene–cell type associations is currently not feasible. Therefore, our findings should be considered hypothesis-generating and await validation in future BCC-specific sc-eQTL cohorts.

Together, these findings from our exploratory PBMC-based scTWAS provide a hypothesis-generating framework for understanding how BCC-associated GWAS variants may influence immune-related pathways in circulating immune cells. However, given that our analyses are based on healthy donor PBMCs rather than BCC lesions, future studies using BCC-derived single-cell eQTL data will be necessary to validate whether these programs operate within the tumor microenvironment.

## Supporting information

S1 Fig Gene Ontology enrichment of BCC-associated genes in B_Mem_, CD4_NC_ and CD4_SOX4_ (FDR < 0.05). (DOCX)

S2 Fig Gene Ontology enrichment of BCC-associated genes in CD8_ET_, CD8_NC_ and CD8_S100B_(FDR < 0.05). (DOCX)

S3 Fig Gene Ontology enrichment of BCC-associated genes in DC, Mono_NC_ and Plasma(FDR < 0.05). (DOCX)

S1 Table Top 10 Genomic Loci Contributing to BCC Heritability. (XLSX)

S2 Table Top 20 Genes Associated with BCC from FUSION. (XLSX)

S3 Table The exact number of genes retained for Stage II analysis in each cell type. (XLSX)

## Abbreviations

BCC: Basal cell carcinoma
UV: Ultraviolet
GWAS: Genome-wide association studies
TWAS: Transcriptome-wide association studies
eQTLs/mQTLs: Expression and methylation quantitative trait loci
scRNA-seq: Single-cell RNA sequencing
sc-eQTL: Single-cell expression quantitative trait locus
scTWAS: Single-cell transcriptome-wide association study
FDR: False discovery rate
MONO_C_: Classical monocytes
DAMPs: Damage-associated molecular patterns
QC: Quality control
hm3: HapMap3
PBMCs: Peripheral blood mononuclear cells
MAF: Minor allele frequency
GReX: Genetically regulated expression
CD4_ET_: CD4^+^ effector memory T cells
CD4_NC_: CD4^+^ naive and central memory T cells
CD4_SOX4_: CD4^+^ T cells expressing SOX4
CD8_NC_: CD8^+^ naive and central memory T cells
CD8_S100B_: CD8^+^ T cells with expression of S100B
CD8_ET_: CD8^+^ effector memory T cells
B_Mem_: Memory B cells
B_IN_: Immature and naive B cells
Plasma: Plasma cells
Mono_NC_: Nonclassical monocytes
DC: Dendritic cells
LDSC: LD Score Regression
LAVA: Local Analysis of [co]Variant Association
GeneStart: Gene’s transcription start site
GeneEnd: Gene’s transcription end site
ORA: Over-representation analysis
GO: Gene Ontology
