# Supplementary material for: Leveraging expression quantitative trait loci information in single-cell resolution to identify cell-specific genes for Basal cell carcinoma
Source: PLoS One. 2026 Aug 4;21(8):e0354887. doi: 10.1371/journal.pone.0354887 (PMC13436695; doi:10.1371/journal.pone.0354887)
Supplement: Supplementary file 2 [file pone.0354887.s002.docx]

Supplementary Figure S2. Gene Ontology enrichment of BCC-associated genes in CD8_ET_, CD8_NC_ and CD8_S100B_(FDR < 0.05).
