# Supplementary material for: Leveraging expression quantitative trait loci information in single-cell resolution to identify cell-specific genes for Basal cell carcinoma
Source: PLoS One. 2026 Aug 4;21(8):e0354887. doi: 10.1371/journal.pone.0354887 (PMC13436695; doi:10.1371/journal.pone.0354887)
Supplement: Supplementary file 3 [file pone.0354887.s003.docx]

Supplementary Figure S3. Gene Ontology enrichment of BCC-associated genes in DC, Mono_NC_ and Plasma(FDR < 0.05).
